# Applying Tissue and Mesh Combined Repair (TMC Repair) to Treat Adult Inguinal Hernia—A Study Based on 1,169 Cases

**DOI:** 10.3389/fsurg.2021.810212

**Published:** 2022-01-25

**Authors:** Lung-Fei Chen

**Affiliations:** Lung-Fei Hernia Hospital, Tainan, Taiwan

**Keywords:** tissue and mesh combined repair, adult inguinal hernia, tissue repair, mesh repair, hernia repair

## Abstract

At present, the most widely favored technique for treating adult inguinal hernia is the use of tension-free mesh repair, while traditional means of tissue repair have been gradually phased out. Mesh repair seeks to prevent the occurrence of hernias by covering the entire myopectineal orifice with artificial mesh. Yet in doing so, it fails to reinforce the naturally weak area between the transversus abdominis arch and the iliopubic tract. To rectify this and reduce the chance of recurrence following inguinal hernia surgery, the author has combined mesh repair with autologous tissue repair to create a method called TMC repair (tissue and mesh combined repair). From Jan 1, 2010 to Dec 31, 2015 the author applied TMC repair to treat adult inguinal hernia in 1,169 patients, achieving a recurrence rate of 0.68%. It is therefore a highly recommended method for treating adult inguinal hernia.

## Background

Surgical methods for treating adult inguinal hernia are numerous and varied, and can be broadly divided into two categories based on the material used: autologous tissue or artificial mesh. Treatments involving autologous tissue are referred to as “tissue repair” and have become less and less common over time (1), while treatments that implant mesh patches fall under the generic umbrella of “mesh repair.”

Prominent surgical methods in the area of tissue repair include the Bassini repair technique (2), iliopubic tract repair (3), Cooper's ligament repair (4), and Shouldice repair (5).

On the other hand, oft-cited mesh repair methods include the use of polyethylene mesh patches to repair defects in the abdominal wall, as espoused by Usher in 1959 (6), the use of large Dacron patches by Stoppa in 1967 (7), the Lichtenstein tension-free mesh repair method published in 1989 (8), and the gradual push toward the current trend of laparoscopic mesh hernia repair (1).

## The Evolution of the Author's Inguinal Hernia Operation Method

In treating adult inguinal hernia, the author initially adopted the Bassini repair technique. Yet after undergoing training in 1988 at Canada's Shouldice Hospital, the author started to apply the Shouldice technique (9) and, from 1996, slowly began to combine this method with the use of artificial mesh to create the TMC repair method.

## The TMC Repair Method

From Jan 1, 2010 to Dec 31, 2015, the author applied TMC repair to treat a total of 1,169 adult inguinal hernia patients. This group of patients consisted of 1,107 males and 62 females; 1,067 first-time sufferers and 102 recurring hernia sufferers (all post primary open repairs), while the types of hernias included 348 direct inguinal hernias, 794 indirect inguinal hernias, 12 combined inguinal hernias, and 15 sliding inguinal hernias. The age distribution of patients ranged from 18 to 96, with a median age of 59. The average time of surgery was 29 and 35 min for first-time and recurring sufferers, respectively.

**Figure 1:**
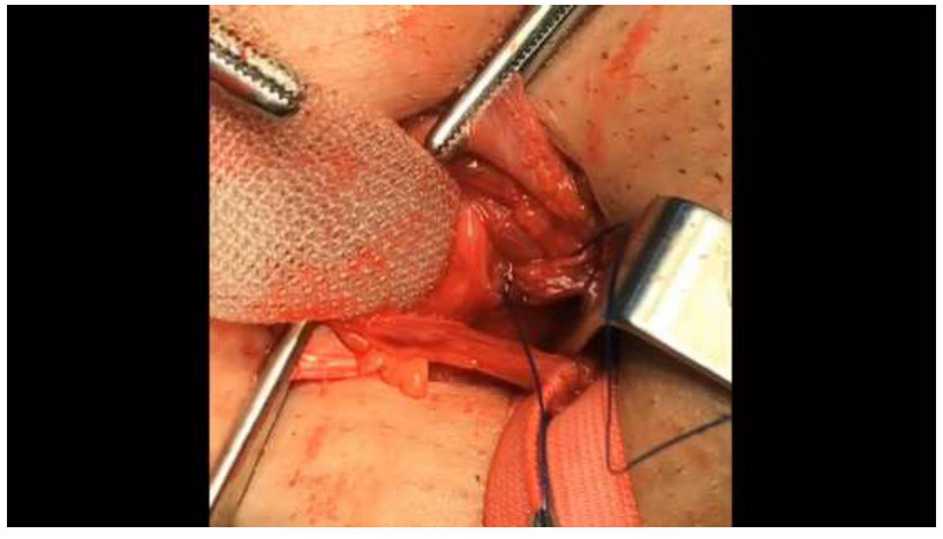
The first thread of the needle should fixate the mesh to the area beneath the transversalis fascia in the external inguinal ring. The lower edge of the mesh should extend to at least 4 cm below the superior of the pubic bone.

**Figure 2:**
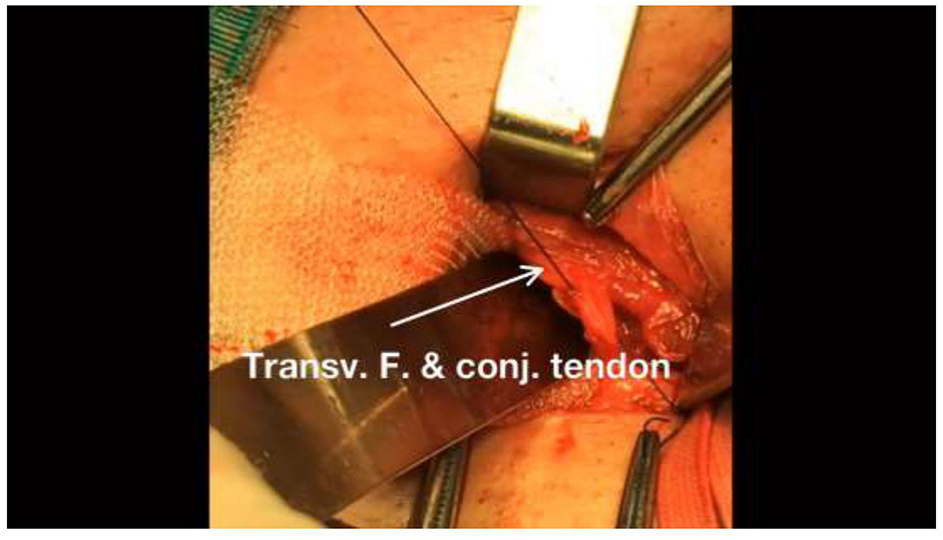
Continue threading the same line of sutures to the internal transversalis fascia and conjoint tendon.

**Figure 3:**
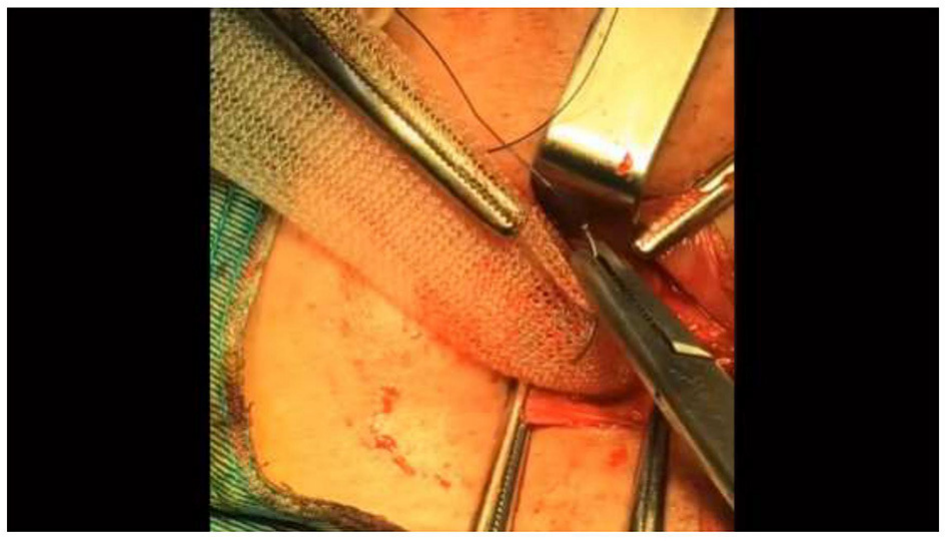
Run the hook through the center of the mesh.

**Figure 4:**
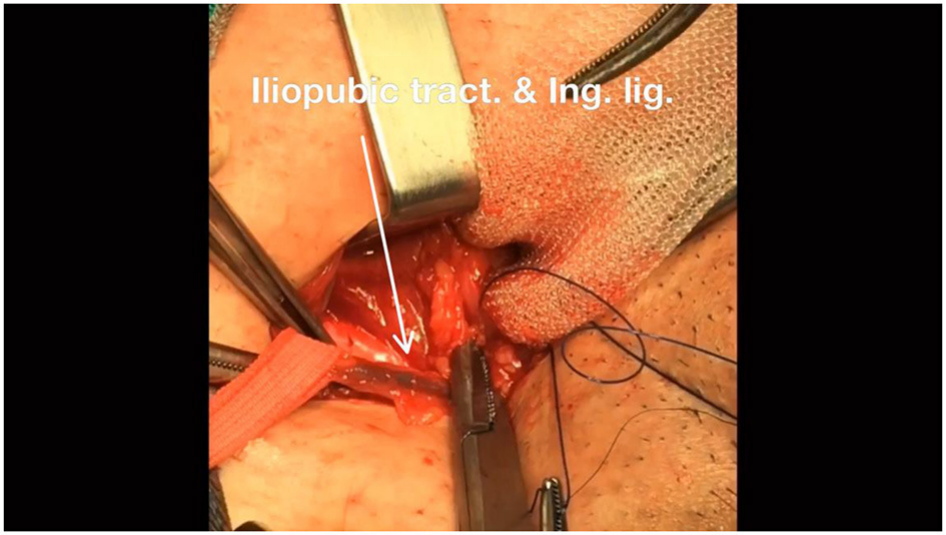
Then, continue suturing to the external iliopubic tract or above the inguinal ligament.

**Figure 5:**
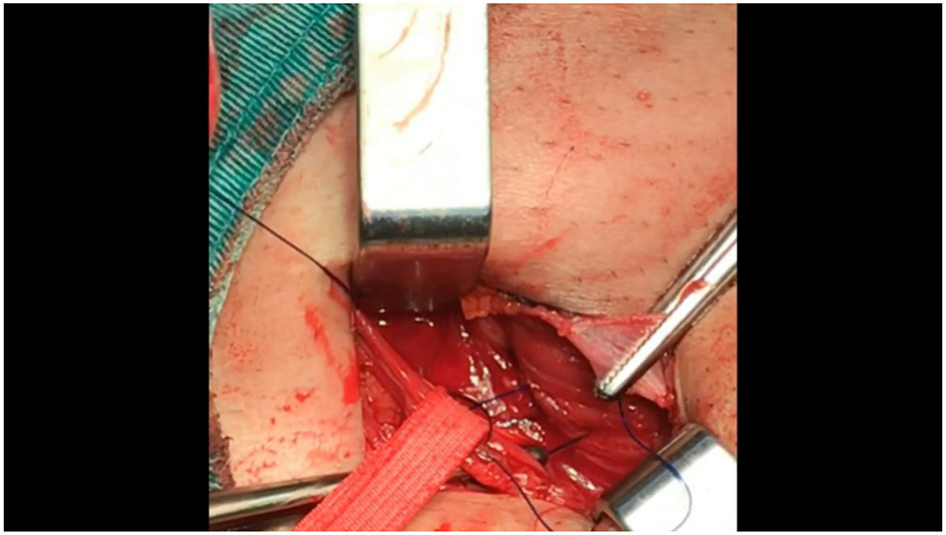
After reconstructing the internal ring (with the mesh already embedded in the preperitoneal space), the same line of suturing should be threaded back toward the external ring to create a continuous suture.

The TMC repair method, created and used by the author, comprises the following steps:

- Make a transverse incision in the lower abdominal wall between the pubic tubercle and the anterior superior iliac spine.
- Peel back the subcutaneous tissue up to the external oblique aponeurosis to reveal the inguinal ligament and the rectus sheath.
- Inspect to see whether a femoral hernia is present.
- Incise the external oblique aponeurosis from the external inguinal ring to the internal inguinal ring and pull the flaps out to the sides. Be careful to protect the ilioinguinal and iliohypogastric nerves below.
- Isolate the spermatic cord below the external oblique aponeurosis and use tape to keep it separated.
- Open the external spermatic fascia, cremaster muscle, and internal spermatic fascia on the spermatic cord, and search here for an indirect hernia sac, or beneath the transversalis fascia for a direct hernia sac.
- Push the hernia sac back into the abdominal cavity. Incise the transversalis fascia to do a high dissection and prop open the preperitoneal space. The lower edge should extend to at least 4 cm below the superior of the pubic bone; the upper edge should extend past the internal inguinal ring; the medial side should exceed the lateral border of the rectus abdominis muscle, and the lateral side should exceed the iliopubic tract.
- Next, place the mesh in the preperitoneal space and suture it to the tissue. Using a single 2/0 polypropylene thread, create two continuous suture lines by threading the needle back and forth between the internal inguinal ring and the pubic bone.
- The first thread of the needle should fixate the mesh to the area beneath the transversalis fascia in the external inguinal ring (Figure 1).
- The same thread should then form a continuous suture that extends toward the exit of the internal inguinal ring. Suture the internal abdominal oblique, transversus abdominis, and transversalis fascia to the external iliopubic tract; suture in place the underlying mesh up to the internal inguinal ring and then secure the suture to complete the first line. At this point, the entire mesh patch is securely implanted in the preperitoneal space and the internal inguinal ring has been reconstructed anew (Figures 2–4).
- Then, make the second continuous suture line by moving the thread back toward the pubic bone. Once again, suture the internal abdominal oblique and the transversus abdominis to the external iliopubic tract or inguinal ligament. When reaching the pubic bone, secure the suture to complete the second continuous suture line on the posterior abdominal wall (Figure 5).
- Make a continuous suture attaching the external oblique aponeurosis and Scarpa's fascia to the area beneath the spermatic cord.
- Pull the spermatic cord and testis back to their original position, and close the subcutaneous tissue and skin to conclude the surgical process.

## Results

A total of 80% of post-operative patients agreed to undergo tracking and examination for 3–8 years following surgery. The average time of surgery was 29 and 35 min for first-time and recurring sufferers, respectively, and patients were able to stand up and walk immediately after surgery. The chief complaints from patients following surgery included mild scrotal bruising and swelling, as well as wound pain, which can be controlled using regular, orally-administered analgesics.

Of these participants, eight patients experienced a recurrence (0.68%), nine encountered infections (0.7%), and, with the exception of one patient who required the mesh to be removed, all other patients completed their treatment using regular methods of disinfection or debridement. Dressing change and debridement were required for eight patients with hematoma (0.7%) and five patients with seroma (0.4%), but the wounds healed fully after draining with 1–2 pulls of a regular syringe; there were no signs of testicular inflammation, testicular atrophy, or lung complications. The recurrence rate (0.68%) was obtained by means of simple phone interviews.

## Discussion

TMC repair draws its heritage partly from the tissue-to-tissue repair techniques of Shouldice repair, merging these techniques with mesh repair. Subsequent to the Bassini repair method, which was pioneered by the father of modern hernia surgery, the most widely adopted form of tissue repair became Shouldice repair. In actual fact, Shouldice repair incorporates many of Bassini repair's basic principles, such as the suturing of the internal abdominal oblique, transversus abdominis, and transversalis fascia (1). However, from a technical perspective, Shouldice repair employs continuous sutures rather than interrupted sutures, which creates relatively even tissue tension following repair; each suture is solid but not tight, and this lowers the chances of recurrence, reduces dead space, and prevents hematoma. Additionally, if the sutures break or the knots come loose, there is less chance of continued loosening or splitting as the sutures are stuck to the tissue (10). With Shouldice repair, the transversalis fascia is incised and the ensuing flaps are overlapped with sutures, thereby reinforcing the strength of the transversalis fascia (10).

For some elements of tissue repair, TMC repair draws on techniques used in Shouldice repair albeit with minor adjustments added. For instance, TMC repair employs 2/0 polypropylene suture (Prolene.Ethicon.UK) as surgical suture instead of number 34 surgical stainless steel suture used by the Shouldice Hospital itself. In addition, instead of moving two suture threads back and forth to create four suture lines in Shouldice repair, TMC repair uses one suture thread to create two suture lines; the first suture line in TMC repair replaces the first and second suture lines of Shouldice repair, while the second suture line in TMC repair replaces the third and fourth suture lines of Shouldice. It therefore simplifies the surgical process.

TMC repair only requires that all tissue and tissue sutures are close, and does not require that they are tightly packed together. This mitigates tension-based issues that arise during conventional tissue repair. Consequently, in the unfortunate case of a nerve being caught in the suture, it will not be wrapped too tightly and post-surgery nerve pain can be avoided or at least lessened. At the same time, the pull of the sutures in the femoral sheath is weakened, which lowers the chances of developing a femoral hernia following Shouldice repair (11).

**Figure 6:**
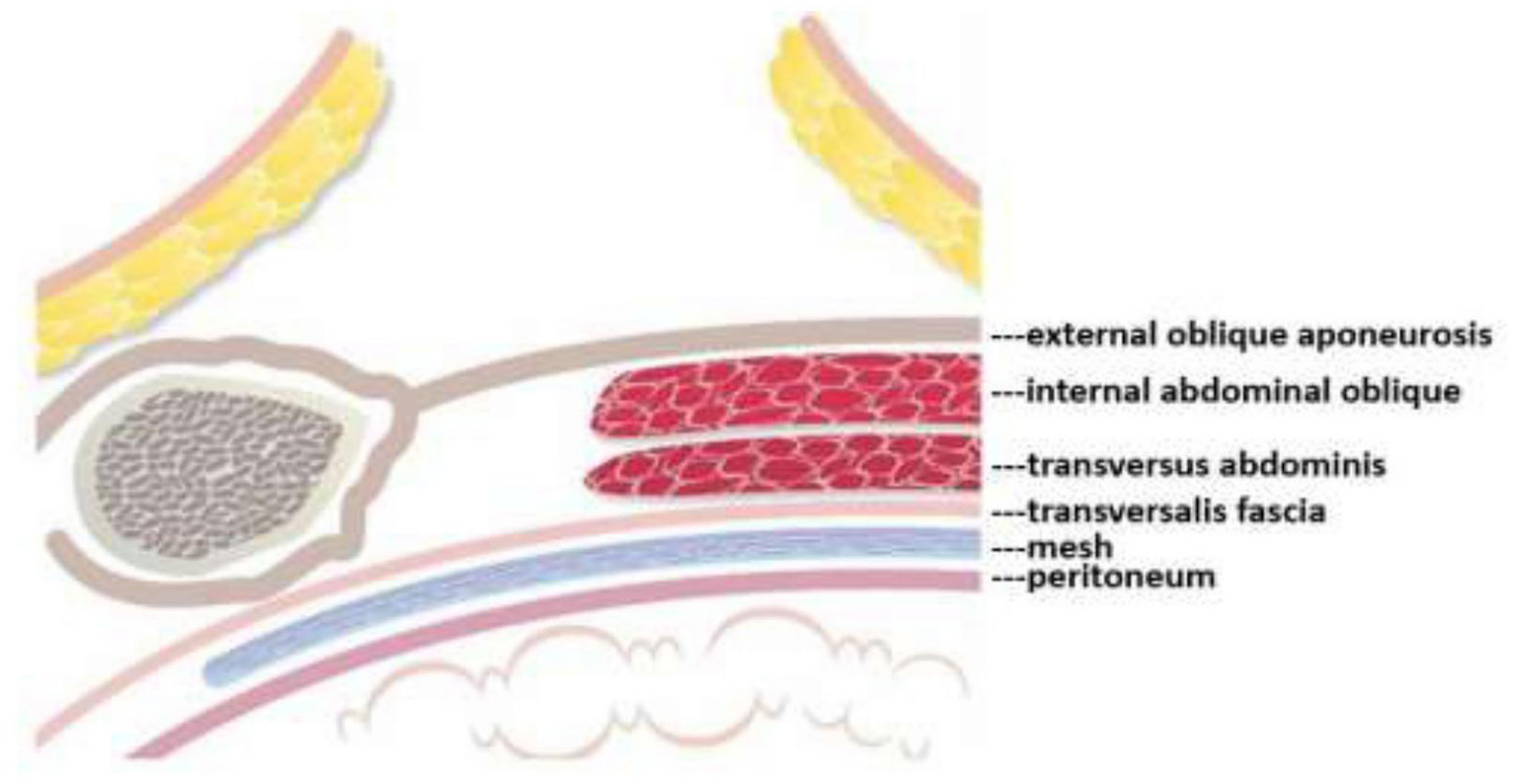
Following mesh repair, the posterior abdominal wall is only protected by a mesh patch and a single layer of autologous tissue—the transversalis fascia.

**Figure 7:**
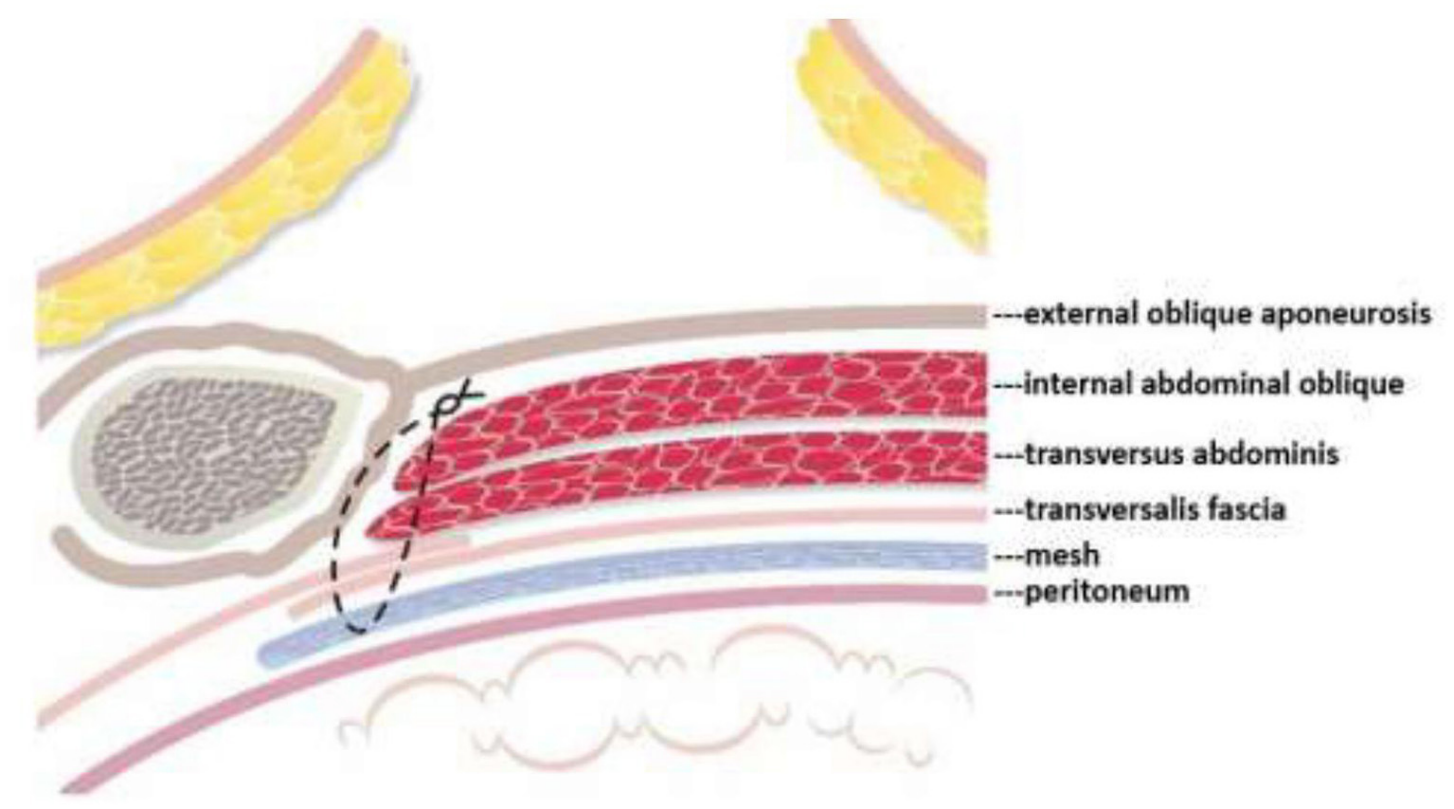
Following TMC repair, aside from the mesh patch itself, the posterior abdominal wall is further protected by a sturdy three layers of autologous tissue—the internal abdominal oblique, transversus abdominis, and the reinforced transversalis fascia.

Mesh repair, on the other hand, consists of covering the myopectineal orifice with a mesh patch, but it fails to repair or strengthen the weak areas in the posterior abdominal wall. To remedy this flaw the author developed the TMC repair method, which not only shields the myopectineal orifice with a mesh patch, but also fortifies autologous tissue in the weak areas of the posterior abdominal wall. This posterior wall originally only comprised one layer of autologous tissue in the form of the transversalis fascia (see Figure 6, below). After TMC repair, however, it becomes a thick triple-layer of autologous tissue comprising the internal abdominal oblique, the transversus abdominis, and the reinforced transversalis fascia, which collectively work to prevent the recurrence of hernia, as highlighted in the Figures 6, 7.

Local anesthetic was administered in all 1,169 cases, and patients could stand up and walk immediately after surgery. Local anesthetic is useful in monitoring the condition of the patient's hernia and potential nerve pain during the operation. It can also increase the chances of surgical success and ease postoperative nerve pain. Moreover, the ability to remain active soon after the operation lowers the risk of experiencing symptoms associated with pneumonia, atelectasis, and pulmonary embolism. None of the patients operated on by the author experienced any such complications, nor were there any instances of subsequent femoral hernias or long-term complications related to chronic pain. Patients could resume light physical exercise after 1 month and heavier, more strenuous activities after 3–6 months.

## Conclusion

The author treats adult inguinal hernia using the TMC repair method, which incorporates the strengths of both mesh and tissue repair. It fortifies the posterior abdominal wall with multiple layers of protection, thereby reducing the chance of post-surgery recurrence, and is characterized by a short operation time and minimal complications. It is thus an excellent surgical option for treating adult inguinal hernia.

## Data Availability Statement

The original contributions presented in the study are included in the article/supplementary material, further inquiries can be directed to the corresponding author/s.

## Ethics Statement

Ethical review and approval was not required for the study on human participants in accordance with the local legislation and institutional requirements. The patients/participants provided their written informed consent to participate in this study.

## Author Contributions

The author confirms being the sole contributor of this work and has approved it for publication.

## Conflict of Interest

The author declares that the research was conducted in the absence of any commercial or financial relationships that could be construed as a potential conflict of interest.

## Publisher's Note

All claims expressed in this article are solely those of the authors and do not necessarily represent those of their affiliated organizations, or those of the publisher, the editors and the reviewers. Any product that may be evaluated in this article, or claim that may be made by its manufacturer, is not guaranteed or endorsed by the publisher.
